# Safety and Efficacy of Vaptans in the Treatment of Hyponatremia from Syndrome of Inappropriate Antidiuretic Hormone Secretion (SIADH): A Systematic Review and Meta-Analysis

**DOI:** 10.3390/jcm12175483

**Published:** 2023-08-24

**Authors:** Pajaree Krisanapan, Supawit Tangpanithandee, Charat Thongprayoon, Pattharawin Pattharanitima, Andrea Kleindienst, Jing Miao, Iasmina M. Craici, Michael A. Mao, Wisit Cheungpasitporn

**Affiliations:** 1 Division of Nephrology and Hypertension, Department of Medicine, Mayo Clinic, Rochester, MN 55905, USA; supawit_d@hotmail.com (S.T.); charat.thongprayoon@gmail.com (C.T.); miao.jing@mayo.edu (J.M.); craici.iasmina@mayo.edu (I.M.C.); 2 Division of Nephrology, Department of Internal Medicine, Faculty of Medicine, Thammasat University, Pathum Thani 12120, Thailand; pattharawin@hotmail.com; 3 Division of Nephrology, Department of Internal Medicine, Thammasat University Hospital, Pathum Thani 12120, Thailand; 4 Chakri Naruebodindra Medical Institute, Faculty of Medicine Ramathibodi Hospital, Mahidol University, Samut Prakan 10540, Thailand; 5 Department of Neurosurgery, Friedrich-Alexander-University Nürnberg-Erlangen, 91054 Erlangen, Germany; andrea.kleindienst@fau.de; 6 Division of Nephrology and Hypertension, Department of Medicine, Mayo Clinic, Jacksonville, FL 32224, USA; mao.michael@mayo.edu

**Keywords:** hyponatremia, SIAD, SIADH, syndrome of inappropriate antidiuresis, syndrome of inappropriate antidiuretic hormone secretion, tolvaptan, satavaptan, vaptans, vasopressin receptor antagonist, V2R antagonists

## Abstract

The utilization of vasopressin receptor antagonists, known as vaptans, in the management of hyponatremia among patients afflicted with the syndrome of inappropriate antidiuretic hormone (SIADH) remains a contentious subject. This meta-analysis aimed to evaluate the safety and efficacy of vaptans for treating chronic hyponatremia in adult SIADH patients. Clinical trials and observational studies were identified by a systematic search using MEDLINE, EMBASE, and Cochrane Database from inception through September 2022. The inclusion criteria were the studies that reported vaptans’ safety or efficacy outcomes compared to placebo or standard therapies. The study protocol was registered with the International Prospective Register of Systematic Reviews (PROSPERO; CRD 42022357307). Five studies were identified, comprising three RCTs and two cohort studies, enrolling a total of 1840 participants. Regarding short-term efficacy on days 4–5, vaptans exhibited a significant increase in serum sodium concentration from the baseline in comparison to the control group, with a weighted mean difference of 4.77 mmol/L (95% CI, 3.57, 5.96; I^2^ = 34%). In terms of safety outcomes, the pooled incidence rates of overcorrection were 13.1% (95% CI 4.3, 33.6; I^2^ = 92%) in the vaptans group and 3.3% (95% CI 1.6, 6.6; I^2^ = 27%) in the control group. Despite the higher correction rate linked to vaptans, with an OR of 5.72 (95% CI 3.38, 9.70; I^2^ = 0%), no cases of osmotic demyelination syndrome were observed. Our meta-analysis comprehensively summarizes the efficacy and effect size of vaptans in managing SIADH. While vaptans effectively raise the serum sodium concentration compared to placebo/fluid restriction, clinicians should exercise caution regarding the potential for overcorrection.

## 1. Introduction

Hyponatremia is a widely acknowledged risk factor for increased mortality, morbidity, and hospital length of stay [1,2,3,4,5,6,7,8]. In addition, chronic hyponatremia is linked to mental impairment, abnormal gait, falls, osteoporosis, and a higher risk of fractures [9,10,11,12,13]. The syndrome of inappropriate antidiuretic hormone secretion (SIADH) is recognized as one of the most common causes of hyponatremia in hospitalized patients [14,15,16]. In a large multinational hyponatremia registry, the prevalence of SIADH was reported to be as high as 49% in euvolemic and hypervolemic patients [17]. The pathophysiology of SIADH is characterized by the inappropriate reduction of free water clearance due to elevated levels of plasma vasopressin (AVP) or gain-of-function activity of the V2 vasopressin receptor (V2R). SIADH can be caused by various underlying disorders, such as malignancy, pulmonary disorders, central nervous system disorders, HIV infection, or idiopathic [18,19,20].

Vaptans, or V2R antagonists, are a novel class of drugs that can effectively treat hyponatremia by directly inhibiting V2R [21,22,23,24]. Several vaptans have been developed for clinical use, including conivaptan, lixivaptan, satavaptan, and tolvaptan. Currently, however, only tolvaptan and conivaptan have been approved by the Food and Drug Administration (FDA) for the treatment of clinically significant hypervolemic and euvolemic hyponatremia [25] and solely tolvaptan has received authorization from the European Medicines Agency (EMA) for treating SIADH. Even though tolvaptan showed significant short-term efficacy in increasing serum sodium in the Study of Ascending Levels of Tolvaptans in Hyponatremia (SALT-1 and SALT-2) [16], the use of vaptans as first-line, or even second-line, therapy among SIADH patients remains controversial [2,26,27,28,29].

To date, two meta-analyses published in 2010 and 2011 have reported the short-term efficacy and safety of vaptans in comparison to placebo for the treatment of hyponatremia. These studies examined hyponatremia not specific to the etiology of SIADH [25,30]. Overall, vaptans modestly increased the serum sodium concentration after 3–7 days, but there was a 2.5 to 3 times increased risk of an overly rapid increase in the serum sodium concentration [2,25,30]. Thus, the European guideline recommends against the use of vaptans for SIADH treatment due to the risk of overcorrection, where the primary concern is for the development of osmotic demyelination syndrome (ODS) [2,31,32]. Moreover, vaptans are expensive and their use in symptomatic patients has yet to be evaluated [21]. Nevertheless, recently, there has been a global urea shortage, resulting in a concern related to the availability of urea treatment for SIADH in some countries. Therefore, it is worth investigating if vaptans could become an alternative therapy for SIADH.

The safety and efficacy of vaptans in the treatment of hyponatremia from SIADH remain undetermined. Therefore, we conducted a systematic review and meta-analysis to evaluate the safety and short-term efficacy of vaptans versus placebo or standard treatment for patients with SIADH.

## 2. Materials and Methods

### 2.1. Search Strategy and Study Eligibility

The protocol for this systematic review and meta-analysis was registered with the International Prospective Register of Systematic Reviews (PROSPERO; CRD42022357307). Two investigators (P.K. and S.T.) independently performed a systematic search of MEDLINE, EMBASE, Cochrane Central Register of Controlled Trials, and Cochrane Database of Systematic Reviews from inception through September 2022. To assess the safety and efficacy of vaptans in the treatment of hyponatremia from SIADH, the search terms included “Vaptans OR tolvaptan OR conivaptan OR satavaptan OR lixivaptan” and “syndrome of inappropriate antidiuretic hormone secretion OR SIADH”. The search strategy is provided in the Supplementary Table S1. The search was limited to humans without language restrictions. A manual search of the references of the included studies for additional relevant studies was also conducted. The reporting of this systematic review followed the standards of the PRISMA Statement [33].

Studies were included in this meta-analysis if they were clinical trials or cohort studies that reported the safety or efficacy outcomes of vaptans (any dosage regimen) in comparison to placebo or any standard therapies (including fluid restriction, urea, salt tablet, and furosemide) for the treatment of hyponatremia in adult SIADH patients (age ≥ 18 years), who were either hospitalized or nonhospitalized. Eligible studies needed to provide at least one of the following outcomes: the early change in serum sodium level from the baseline (at day 2–7), the rate of response, or the rate of sodium level overcorrection.

Studies that primarily reported other treatment outcomes or were comprised of mixed etiologies of hyponatremia without a subgroup analysis for SIADH alone were also excluded. Retrieved studies were independently reviewed for eligibility by the two investigators (P.K. and S.T.). Discrepancies were resolved by mutual consensus between all authors.

### 2.2. Data Extraction and Quality Assessment

A standardized data collection form was used to collect the following from each included study: study title, author names, publication year, study type, the country where the study was conducted, number of patients, age, follow-up duration, inclusion criteria, causes of SIADH, control, study drug, dose of vaptans, drug exposure, interested outcomes, baseline serum sodium, efficacy outcomes, days of efficacy assessment, change of serum sodium from the baseline, the proportion of responders, time to response, overcorrection, need for rescue therapy, mortality, adverse events, and withdrawn due to adverse event. The outcome of ODS was primarily defined by the original included studies. 

The quality of each study was independently appraised by each investigator. The Newcastle–Ottawa quality scale (NOS) was used for cohort studies [34] (Supplementary Table S2), and the Cochrane risk of bias tool was used to assess the quality of RCTs (Supplementary Figure S1) [35].

### 2.3. Statistical Analysis

A comprehensive meta-analysis version 3.3.070 (Biostat Inc., Englewood, NJ, USA) was used to perform this meta-analysis. The differences in effects were expressed as odds ratios with 95% confidence intervals (CIs) for dichotomous outcomes and the weighted mean difference (WMD) with 95% confidence intervals for continuous outcomes. If those data were unavailable in the original articles, we calculated them by using data obtained from investigators or figures. For RCTs, participants were analyzed in the groups of intention to treat.

Heterogeneity was assessed using χ^2^ and/or the I^2^ statistic, with I^2^ values above 50% or a *p*-value below 0.1, indicating significant heterogeneity. In the presence of significant heterogeneity, a random-effect model was applied for meta-analysis. To evaluate the presence of publication bias, a funnel plot and the Egger test [36] were utilized. A subgroup analysis for primary outcomes based on the types of control was planned but could not be performed due to the limited data availability. A sensitivity analysis of overcorrection was performed by excluding a study by Kleindienst et al. [37] given that this study included solely patients with SIADH following postoperative pituitary surgery. A *p*-value < 0.05 was considered statistically significant for all analyses.

## 3. Results

### 3.1. Study Characteristics

**Figure 1:**
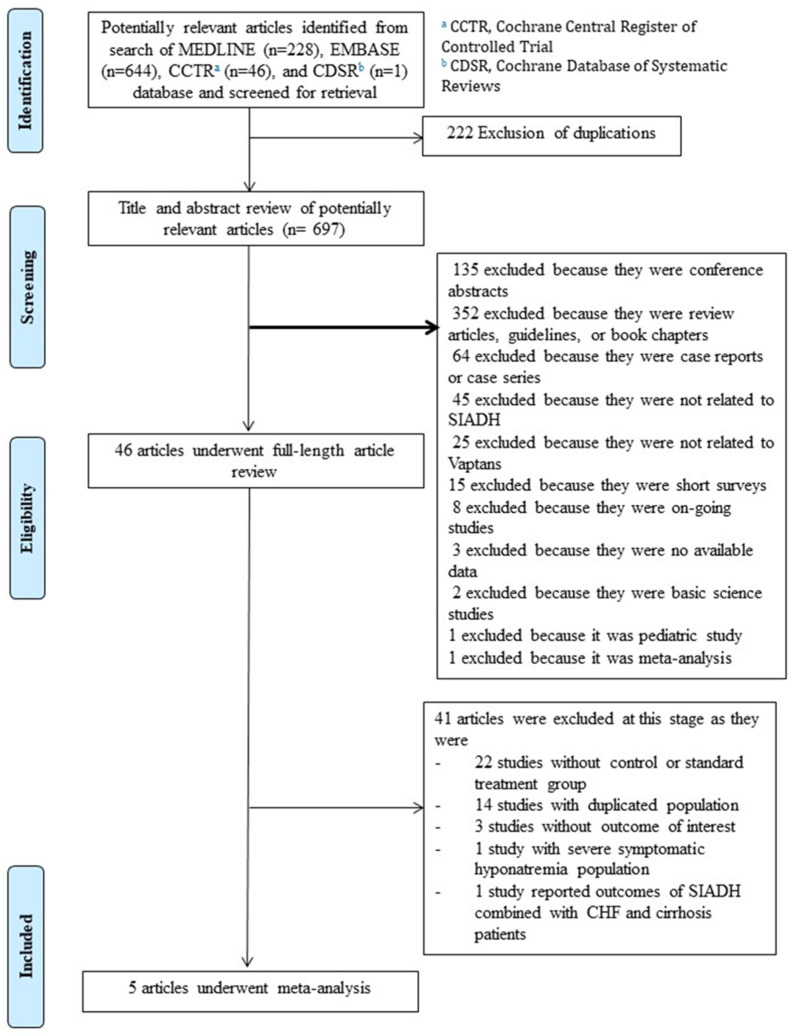
PRISMA flow diagram.

A total of 919 potential articles were retrieved by our search strategy (Figure 1), of which 222 were duplicated articles. We excluded 651 articles because they did not meet the inclusion criteria due to article type, methodology, or lack of relevance to both vaptans and SIADH. Therefore, 46 articles underwent full-length review. Forty-one articles were further excluded because of duplicated population, lack of controls, lack of outcome of interest, no subgroup analysis of SIADH patients, or it included a population with severe symptomatic hyponatremia. Only five studies with a total of 1840 patients were included in this systematic review and meta-analysis, consisting of three RCTs [37,38]. and two cohort studies [37,38].

Tolvaptan was used in most of the studies with a dose ranging from 3.75 to 60 mg once daily [37,38,39,40], whereas only one study investigated satavaptan [41] (Table 1). The control or standard treatment group consisted of a placebo in three RCTs [39,40,41] and fluid restriction in two cohort studies [37,38]. The median follow-up time was 22.5 days (IQR 7.5, 201), ranging from 7 days to 1 year. The median drug exposure time was 17.5 days (IQR 4, 29), ranging from 1 day to 30 days.

The majority of the patients included in this systematic review were from Europe [37,38,41] and the United States [38,40], while only a few were from China [39]. SIADH etiologies, which were reported in three studies [37,38,41], included idiopathic, malignancy related, drug induced, and postoperative. The latter was highlighted only in Kleindienst et al. since they exclusively evaluated SIADH patients following pituitary tumor surgery [37]. Overall, the mean age of patients was 59.3 ± 15.1 years old in four studies [37,39,40,41]. The mean baseline serum sodium was 127.2 ± 4.3 mmol/L [37,39,41] in three studies and a separate RCT reported that half of the patients had a baseline serum sodium < 130 mmol/L [40].

### 3.2. Efficacy of Vaptans on the Changes in Serum Sodium Levels from Baseline

**Figure 2:**
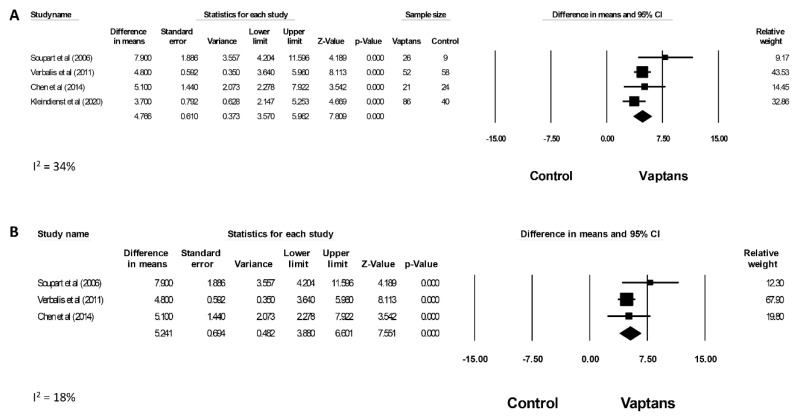
Alterations in Serum Sodium Levels from the Baseline. (**A**) Early change in serum sodium concentration from the baseline contrasting vaptans to the control, based on meta-analysis results; Soupart et al. [41]; Verbalis et al [40]; Chen et al. [39] and Kleindienst et al. [37]. (**B**) Sensitivity analysis considering only RCTs, still indicating significant change from the baseline when compared to the control. For both analyses, studies are referenced by the initial author’s name and publication year. Soupart et al. [41]; Verbalis et al [40] and Chen et al. [39]. Weighted mean differences were consolidated utilizing the random-effects model, depicted on a scale ranging from −15 to 15 mEq/L. Abbreviation: CI-confidence interval [39,40,41]. Squares: Represent the point estimate of the effect from individual studies. The size of the square often reflects the weight of the study in the meta-analysis, with larger squares indicating greater weight. Diamond: Represents the summarized effect estimate from the combined studies. The width of the diamond provides an idea about the precision of the estimate; a wider diamond suggests less precision, while a narrower diamond indicates more precision.

Four studies assessed the changes in serum sodium levels from the baseline, with three studies reporting changes on day 4 [37,39,40] and one study reporting changes on day 5 [41]. The reported changes in serum sodium were presented as either absolute values or average daily area under the curve (AUC) (Supplementary Table S3). In the meta-analysis, the use of vaptans demonstrated a significant increase in the change of serum sodium levels from baseline compared to the control group (placebo/fluid restriction), with a WMD of 4.77 mmol/L (95% CI, 3.57, 5.96; I^2^ = 34%) (Figure 2A). Additionally, a sensitivity analysis was additionally performed including only RCTs. A significant increase in the change of serum sodium levels from the baseline compared to the control group remained significant, with a WMD of 5.24 mmol/L (95% CI, 3.88, 6.60; I^2^ = 18%) (Figure 2B).

### 3.3. Efficacy of Vaptans on the Response of Treatment

**Figure 3:**
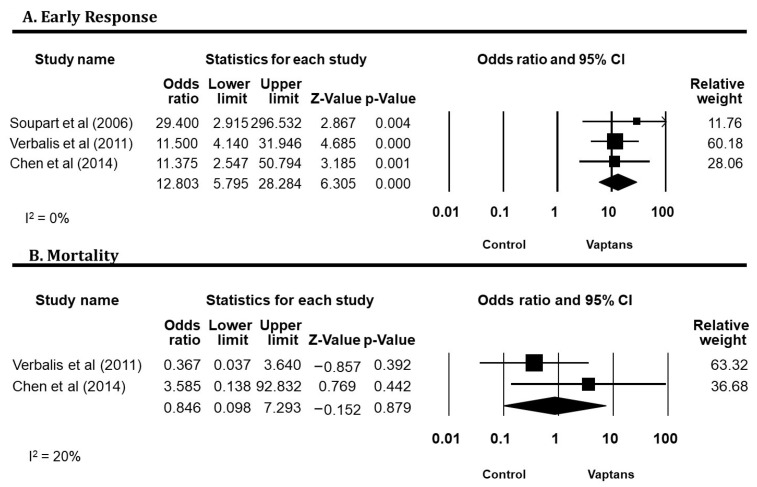
Pooled odds ratio of early response (**A**) (Soupart et al. [41]; Verbalis et al [40]; Chen et al. [39,40,41] and mortality (**B**) (Verbalis et al [40]; Chen et al. [39,40,41] comparing vaptans with the control. Studies were identified by the name of the first author and the year of publication. Odds ratios were pooled using the random-effects model and shown on a scale of 0.01–100. Abbreviation: CI: confidence interval. Squares: Represent the point estimate of the effect from individual studies. The size of the square often reflects the weight of the study in the meta-analysis, with larger squares indicating greater weight. Diamond: Represents the summarized effect estimate from the combined studies. The width of the diamond provides an idea about the precision of the estimate; a wider diamond suggests less precision, while a narrower diamond indicates more precision.

The early response after vaptan treatment at days 4–5 was assessed in three studies [38,39,40]. The responders were defined as patients whose serum sodium normalized (135 mmol/L) or increased by at least 5 mmol/L from the baseline. Overall, there were greater numbers of responders in the vaptans with a pooled odds ratio (OR) of 12.80 (95% CI 5.78, 28.28; I^2^ = 0%) (Figure 3A).

### 3.4. Efficacy of Vaptans on Mortality

Mortality was reported in two RCTs consisting of 154 patients [39,40]. Overall, five deaths were reported without a significant difference between the two treatment groups (OR 0.85; 95% CI 0.10, 7.29; I^2^ = 20%) (Figure 3B).

### 3.5. Safety of Vaptans on Overcorrection

**Figure 4:**
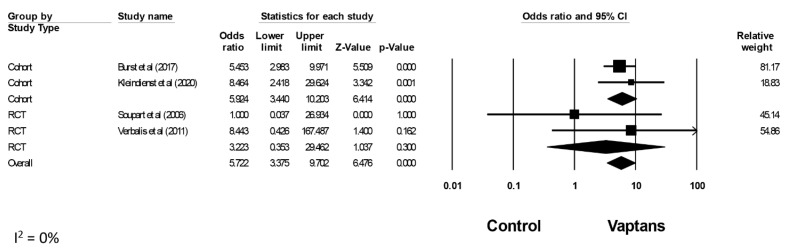
Pooled odds ratio of overcorrection with vaptans compared to the control. Studies were identified by the name of the first author, year of publication, and type of study. Odds ratios were pooled using the random-effects model and shown on a scale of 0.01–100. Abbreviation: CI: confidence interval (Burst et al [38]; Soupart et al. [41]; Verbalis et al [40]; Kleindienst et al. [37]). Squares: Represent the point estimate of the effect from individual studies. The size of the square often reflects the weight of the study in the meta-analysis, with larger squares indicating greater weight. Diamond: Represents the summarized effect estimate from the combined studies. The width of the diamond provides an idea about the precision of the estimate; a wider diamond suggests less precision, while a narrower diamond indicates more precision.

There were four studies that assessed serum sodium overcorrection [37,38,40,41]. Mostly, overcorrection was defined as an increase in the serum sodium level > 12 mmol/L over the first 24 h and >18 mmol/L over the first 48 h in three studies [38,40,41], whereas another study had a lower threshold for overcorrection defined as an increase in the serum sodium level >10 mmol/L over the first 24 h [37] (Supplementary Table S4). In this meta-analysis, the pooled incidence rates of overcorrection were 13.1% (95% CI 4.3, 33.6; I^2^ = 92%) in the vaptans group (Supplementary Figure S2) and 3.3% (95% CI 1.6, 6.6; I^2^ = 27%) in the control group (Supplementary Figure S3). Overall, there was a statistically significant increased rate of overcorrection with vaptans with a pooled OR of 5.72 (95% CI 3.38, 9.70; I^2^ = 0%) (Figure 4). Meta-analyses, based on the type of study, were additionally performed. Subgroup analysis by study design revealed a statistically significant increased rate of overcorrection with vaptans in cohort studies with a pooled OR of 5.92 (95% CI 3.44, 10.20; I^2^ = 0%). However, when solely including RCTs, this statistically significant increased risk of overcorrection with vaptans was not observed with a pooled OR of 3.22 (95% CI 0.35, 29.46; I^2^ = 0%).

Sensitivity analysis of overcorrection was performed by excluding a study by Kleindienst et al. [37] given that this study included solely patients with SIADH following postoperative pituitary surgery and had a different definition of overcorrection among the rest of the studies. The pooled incidence rates of overcorrection were 9.2% (95% CI 4.9, 16.9; I^2^ = 34%) in the vaptans group (Supplementary Figure S4) and 2.5% (95% CI 1.6, 3.9; I^2^ = 0%) in the control group (Supplementary Figure S5). Overall, there was a statistically significant increased rate of overcorrection with vaptans with a pooled OR of 5.26 (95% CI 2.94, 9.41; I^2^ = 0%) (Supplementary Figure S6).

### 3.6. Adverse Events with Vaptans

Overall, adverse events requiring drug discontinuation were not significantly different between the two groups (OR 0.90; 95% CI 0.32, 2.53; 3 studies; I^2^ = 0%) (Supplementary Figure S7). However, vaptans were significantly associated with an increased risk of thirst (OR 2.38; 95% CI 1.03, 5.53; three studies; I^2^ = 0%) (Supplementary Figure S8). There was only one RCT that reported hypernatremia with an incidence of 7.69% (2/26) in the vaptan group (satavaptan) and 0% (0/9) in the control group [41].

Interestingly, vaptans were not significantly associated with urinary frequency/polyuria (1.41; 95% CI 0.40, 5.00; two studies; I^2^ = 0%) (Supplementary Figure S9), dry mouth (OR 2.06; 95% CI 0.87, 4.85; two studies; I^2^ = 0%) (Supplementary Figure S10), or hypotension (OR 0.73; 95% CI 0.17, 3.06; two studies; I^2^ = 19%) (Supplementary Figure S11), as compared to the control. Furthermore, across four studies, there were no reported cases of osmotic demyelination syndrome (ODS) [37,38,40,41].

### 3.7. Evaluation of Publication Bias

A funnel plot to assess for publication bias in the difference of mean change in serum sodium from the baseline is shown in Supplementary Figure S12 and for log odds ratio of overcorrection in Supplementary Figure S13. Egger’s regression asymmetry test was utilized and this showed no publication bias with *p* > 0.05 for all analyses.

## 4. Discussion

Our systematic review/meta-analysis is the first study that evaluates the safety and efficacy of vaptans for the treatment of hyponatremia caused exclusively by SIADH. This study demonstrated that vaptans significantly increase serum sodium from the baseline and also increase the rate of early response compared to control groups. There was an increased risk of overcorrection in the vaptan group; however, there were no reported cases of osmotic demyelination syndrome or increased mortality.

The efficacy outcomes in our analysis were consistent with two previous meta-analyses that evaluated vaptans for the treatment of euvolemic and hypervolemic hyponatremia not specific to SIADH. Rozen-Zvi et al. and Jaber et al. reported that vaptans significantly increased the serum sodium level compared to the placebo with a WMD of 5.27 mmol/L (95% CI 4.27, 6.26) at 3–7 days and 5.70 mmol/L (95% CI 4.10, 7.40) at 5 days, respectively [30,42]. Since vaptans directly antagonize the V2R, and SIADH is characterized by inappropriate elevations of AVP or gain-of-function of the V2R, it is not unexpected that vaptans have the highest efficacy in increasing serum sodium compared to the other available therapies for SIADH treatment [38]. These meta-analyses, in sum, reveal that vaptans are effective in both SIADH and other etiologies of hyponatremia.

Although there is evidence that hyponatremia increases the risk of death [1,7,8], our meta-analysis, as well as prior meta-analyses [30,42], did not find that correction of hyponatremia with vaptans reduces mortality. The lack of mortality benefit could be due to a lack of power to detect outcomes, as only 5 deaths out of 154 participants were reported in our eligible studies. Another possible explanation is that SIADH is frequently a consequence of other chronic illnesses, particularly several types of cancers. Malignancy has been reported to be the cause of SIADH in up to 21% of a hyponatremia registry [38]. Thus, the primary disease causing SIADH may override any mortality benefit of hyponatremia correction with vaptans.

It is noted that the rate of overcorrection in our review is significantly higher than in prior meta-analyses, as we found that the risk of overcorrection with vaptan treatment for SIADH patients was 5.7 times higher than with traditional therapy. Even after excluding SIADH from postoperative pituitary surgery in the sensitivity analysis, the risk of overcorrection with vaptan still remained 5.3 times higher than with traditional therapy. Meanwhile, the risk of overcorrection in euvolemic/hypervolemic hyponatremic individuals treated with vaptans was only 2.5 and 3.0 times higher than the placebo [30,42]. Despite the increased risk of overcorrection associated with vaptans, neither our study nor prior meta-analyses found any cases of ODS [30,42].

This increased risk of overcorrection with hyponatremia due to SIADH compared to other hyponatremic etiologies may reflect the greater efficacy of vaptan therapy in SIADH. Jaber et al. reported that significantly greater increases in net serum sodium levels occurred on day 1 with vaptan treatment in euvolemic hyponatremia (i.e., SIADH) compared to those with hypervolemic hyponatremia (i.e., heart failure or cirrhosis) [30]. Our hypothesis for the higher rate of overcorrection in our study could be due to the vaptan dosage. It has been suggested that the approved standard doses of tolvaptan may be excessive for the treatment of SIADH, particularly in patients with very low serum sodium concentrations [43,44,45,46,47]. There is evidence that tolvaptan, at a dose of 7.5 mg/day, is as effective as 15 mg in increasing serum sodium and has a lower rate of overly rapid sodium correction in SIADH patients [43,45,48]. However, most of the included studies in our review evaluated a standard higher dose of vaptan, including tolvaptan at the dose of 30–60 mg/day and satavaptan at the dose of 25–50 mg/day [39,40,41]. There was only one study that evaluated a lower dose of tolvaptan at 3.75–7.5 mg/day [37]. Therefore, future studies are required to assess the appropriate starting dose of vaptans in SIADH patients.

Apart from the risk of sodium overcorrection, vaptans did not increase adverse events that required drug discontinuation in our analysis. Thirst was only found to be significantly associated with vaptans, which is consistent with prior meta-analyses [30,42]. Our study thus showed that vaptans did not increase the risk of other adverse events, including dry lips, polyuria/urinary frequency, or hypotension. However, it is noted that there were only a few studies with a small number of participants included in this meta-analysis that evaluated those adverse side effects.

Our systematic review/meta-analysis has some limitations. First, due to the limited number of included studies, we were unable to conduct subgroup analysis regarding vaptan types (tolvaptan vs. satavaptan), or vaptan doses (low dose vs. standard dose). While the observation regarding the potential differences between tolvaptan and satavaptan is crucial, the scarcity of studies directly comparing these agents within the context of SIADH treatment constrained our ability to undertake a separate assessment of their effects and side effects. Nevertheless, the heterogeneity within the findings of our meta-analysis is not notably high. Second, there was variation in the definition of sodium overcorrection among the eligible studies in our meta-analysis. While the European clinical practice guideline on diagnosis and treatment of hyponatremia defines overcorrection as a rapid increase of serum sodium > 10 mmol/L during the first 24 h or >8 mmol/L in any 24 h thereafter [2], only one of four included studies applied that definition in their studies [37]. The three remaining studies [38,40,41] utilized a higher cut-off for the definition of sodium overcorrection; they defined it as an increase in the serum sodium level > 12 mmol/L over the first 24 h, which might have led to an underestimation of the incidence of overcorrection. In order to mitigate this potential imprecision in outcomes, we conducted a sensitivity analysis by excluding the study conducted by Kleindienst et al. [37]. Third, when solely focusing on RCTs, the statistically significant increased risk of overcorrection with vaptans was not found. This, however, may arise from the controlled environmental parameters intrinsic to clinical trials, in contrast to the varied factors prevalent in real-world data. Furthermore, the lack of statistical significance may be attributed to the power inadequacy, highlighted by the broad confidence interval. Fourth, the majority of studies included in our analysis reported only short-term outcomes ranging from a week to a month. Since SIADH is regarded to be a chronic condition, long-term outcomes are needed to assess long-term efficacy, tolerability, and safety. Fifth, the absence of ODS in our review could potentially be attributed to the restricted number of studies included. Therefore, the imperative for a comprehensive large-scale RCT primarily focusing on the safety assessment of vaptans remains evident. Last, all of the studies in our analysis included patients with mild to moderate hyponatremia but patients with severe hyponatremia or those with significant symptoms were excluded. Thus, this study should be applied cautiously to patients with symptomatic or severe hyponatremia.

## 5. Conclusions

Our research provides a comprehensive analysis of the therapeutic potency and magnitude of the effect of vaptans in managing SIADH. Although vaptans demonstrate a notable enhancement in serum sodium concentrations when contrasted with placebo/FR, there is a pronounced likelihood of an excessive correction. However, the risk associated with osmotic demyelination syndrome is not evident.

## Figures and Tables

**Table 1:** Characteristics of the included studies.

Study (Year)	Study Type	Site	Total N	F/U Time	Population	Age (Years)	Baseline Serum Sodium (mmol/L)	Control(s) (n)	Study Drug (n)	Dose of Vaptans	Drug Exposure
Soupart et al. (2006) [41]	RCT	Multicenter (Belgium, Germany, Hungary, and France)	35	12 months	SIADH patients with stable hyponatremia ^a^	68.0 ± 14.3	125.9 ± 5.1	Placebo (*n* = 9)	Satavaptan (*n* = 26)	25 or 50 mg oral once daily	28 days
Verbalis et al. (2011) [40]	RCT	Multicenter (US)	110	37 days	Subgroup analysis of patients with a diagnosis of SIADH from the SALT-1 and SALT-2 trials	64.5 ± 14.5	54% < 130 mmol/L, 46% ≥ 130 mmol/L	Placebo (*n* = 58)	Tolvaptan (*n* = 52)	30–60 mg oral once daily	30 days
Chen et al. (2014) [39]	RCT	Multicenter (China)	45	7 days	Chinese SIADH patients who hospitalized with nonhypovolemic and nonacute hyponatremia	62.1 ± 13.5	126.1 ± 5.6	Placebo (*n* = 24)	Tolvaptan (*n* = 21)	30–60 mg oral once daily	7 days
Burst et al. (2017) [38]	Prospective multinational registry	Multinational register (US, EU)	1524	N/A	SIADH patients with significant hyponatremia ^b^	88% of patients aged > 50 years	N/A	no active therapy, fluid restriction, salt tablets, FR + NSS, demeclocycline, NSS, hypertonic saline (*n* = 1,402) ^c^	Tolvaptan (*n* = 122) ^c^	N/A	N/A
Kleindienst et al. (2020) [37]	Prospective observational study	Single center (Germany)	126	8 days	Post-operative SIADH patients after pituitary surgery, excluding TSH and ACTH deficiency	51.5 ± 16.4	127.9 ± 3.4	Fluid restriction (*n* = 40)	Tolvaptan (*n* = 86)	3.75 or 7.5 mg oral once daily until resolution	43% of tolvaptan group got a single dose

Abbreviations: ACTH: adrenocorticotropic hormone; FR: fluid restriction; F/U: follow-up; NSS: normal saline; SIADH: syndrome of inappropriate secretion of antidiuretic hormone; TSH: thyroid-stimulating hormone. ^a^ Stable hyponatremia was defined as serum Na^+^ increase ≤ 4 mmol/L between two measurements of 24 h apart and serum Na^+^ between 115–132 mmol/L. ^b^ Significant hyponatremia was defined as serum Na ≤ 130 mmol/L. ^c^ Calculated based on the use of tolvaptan by 8% of all patients.

## Data Availability

Data are available upon reasonable request to the corresponding author.

## Supplementary Materials

The following supporting information can be downloaded at: https://www.mdpi.com/article/10.3390/jcm12175483/s1.
