# Supplementary material for: Safety and Efficacy of Vaptans in the Treatment of Hyponatremia from Syndrome of Inappropriate Antidiuretic Hormone Secretion (SIADH): A Systematic Review and Meta-Analysis
Source: J Clin Med. 2023 Aug 24;12(17):5483. doi: 10.3390/jcm12175483 (PMC10488023; doi:10.3390/jcm12175483)
Supplement: Supplementary file 1 [file jcm-12-05483-s001.zip › jcm-2582277-supplementary.pdf]

Supplemental Material

**Table S1**	Search terms
**Table S2**	Summary of Newcastle Ottawa Scale scores for cohort studies
**Figure S1**	Risk of bias summary and graph for included RCTs
**Table S3**	The change of sodium from baseline
**Table S4**	The definition of overcorrection in the included studies
**Figure S2**	Pooled incidence rates of overcorrection with vaptans
**Figure S3**	Pooled incidence rates of overcorrection in the control
**Figure S4**	Sensitivity analysis of pooled incidence rates of overcorrection with vaptans
**Figure S5**	Sensitivity analysis of pooled incidence rates of overcorrection in the control
**Figure S6**	Sensitivity analysis of pooled odds ratio of overcorrection
**Figure S7**	Pooled odds ratio of adverse events requiring vaptan discontinuation
**Figure S8**	Pooled odds ratio of thirst
**Figure S9**	Pooled odds ratio of urinary frequency or polyuria
**Figure S10**	Pooled odds ratio of dry mouth
**Figure S11**	Pooled odds ratio of hypotension
**Figure S12**	Funnel plot of standard error by difference in mean change of serum sodium from baseline
**Figure S13**	Funnel plot of standard error by log odds ratio of overcorrection

Table S1 Search terms

1	tolvaptan
2	conivaptan
3	lixivaptan
4	satavaptan
5	vaptan$
6	1 or 2 or 3 or 4 or 5
7	SIADH
8	SIAD
9	syndrome of inappropriate antidiuretic
10	syndrome of inappropriate antidiuresis
11	7 or 8 or 9 or 10
12	6 and 11
13	Limited 12 to human

Table S2 Summary of Newcastle Ottawa Scale scores for cohort studies

Author (year)	Selection	Comparability	Outcome	Scores
Burst et al. (2017)	****	*0	***	8
Kleindienst et al. (2020)	****	**	***	9

Figure S1 Risk of bias summary and graph for included RCTs

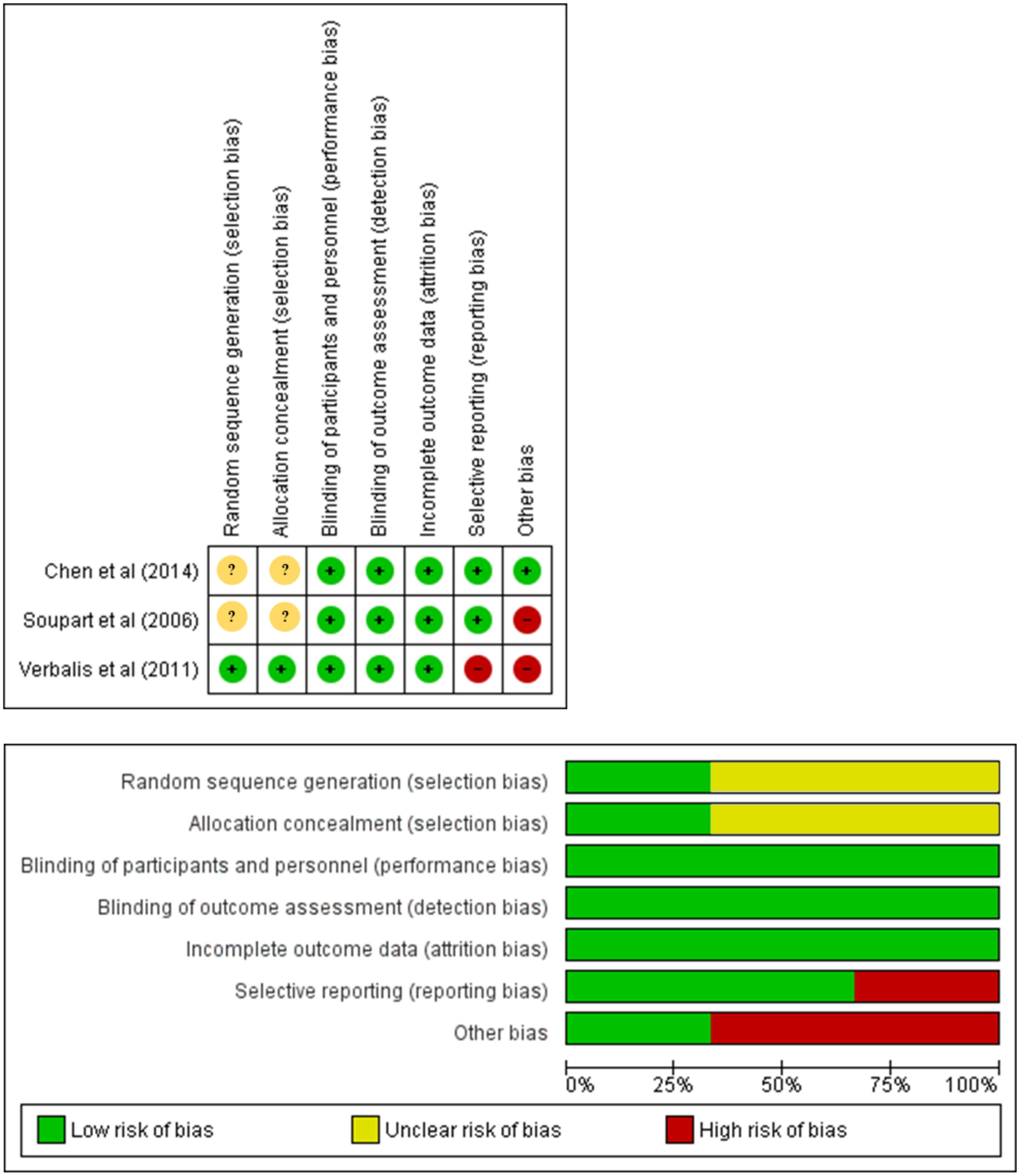

Table S3 The change of sodium from baseline

Study (Year)	Study drug/ control	Reported efficacy outcome (s)	Days of assess ment	Change of serum sodium from baseline (mmol/L)	
				Vaptans	Contol
Soupart et al (2006)	Satavaptan / Placebo	Mean serum sodium at baseline and day 5	5	11.9±5.1 (n=26)	4.0±4.1 (n=9)
Verbalis et al (2011)	Tolvaptan/ Placebo	The change in the average daily AUC for the serum sodium from baseline	4	5.3±3.4 (n=52)	0.5±2.8 (n=58)
Chen et al (2014)	Tolvaptan/ Placebo	The change of the serum sodium from baseline	4	8.4±4.6 (n=21)	3.3±5.0 (n=24)
Kleindienst et al (2020)	Tolvaptan/ Fluid restriction	The change of the serum sodium from baseline	4	8.3±3.8 (n=86)	4.6±4.8 (n=40)

Abbreviations: ACTH, adrenocorticotropic hormone; AUC, area under the curve; FR, fluid restriction; N/A, not applicable; NSS, normal saline;
[a] cancer-related SIADH, [b] non-cancer-related SIADH

Table S4 The definition of overcorrection in the included studies

Study (Year)	Definition of Overcorrection	Overcorrection (%)	
		**Vaptans**	**Control**
**Soupart et al. (2006)**	> 12 mmol/L over the first 24 h and > 18 mmol/L over the first 48 h	3.8 % (1/26)	0% (0/8)
**Verbalis et al. (2011)**	> 12 mmol/L over the first 24 h and > 18 mmol/L over the first 48 h	5.9% (3/51)	0% (0/58)
**Burst et al. (2017)**	> 12 mmol/L over the first 24 h and > 18 mmol/L over the first 48 h	14.1%[a] (10 of 67)	2.9%[b] (4 of 147)
		11.2%[c] (18 of 158)	2.5%[d] (15 of 601)
**Kleindienst et al. (2020)**	> 10 mmol/L over the first 24 h	40.7% (35/86)	7.5% (3/40)

[a] Cancer-related treated with tolvaptan, [b]cancer-related treated with fluid restriction, [c]non-cancer related treated with tolvaptan, [d]non-cancer related treated with fluid restriction

Figure S2 Pooled incidence rates of overcorrection with vaptans

Study name	Event rate	Lower limit	Upper limit	Z-Value	p-Value	Relative weight
Soupart et al (2006)	0.038	0.005	0.228	-3.156	0.002	16.73
Verbalis et al (2011)	0.059	0.019	0.167	-4.659	0.000	23.92
Burst et al (2017)	0.124	0.087	0.174	-9.660	0.000	29.76
Kleindienst et al (2020)	0.407	0.309	0.513	-1.715	0.086	29.59
	0.131	0.043	0.336	-3.064	0.002	

Statistics for each study; Event rate and 95% CI

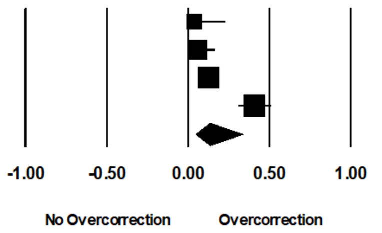

I^2 = 92%

Figure S3 Pooled incidence rates of overcorrection in the control

Study name	Event rate	Lower limit	Upper limit	Z-Value	p-Value	Relative weight
Soupart et al (2006)	0.056	0.003	0.505	-1.947	0.052	6.04
Verbalis et al (2011)	0.008	0.001	0.121	-3.353	0.001	6.31
Burst et al (2017)	0.025	0.016	0.039	-15.695	0.000	61.61
Kleindienst et al (2020)	0.075	0.024	0.208	-4.185	0.000	26.03
	0.033	0.016	0.066	-9.076	0.000	

Statistics for each study; Event rate and 95% CI

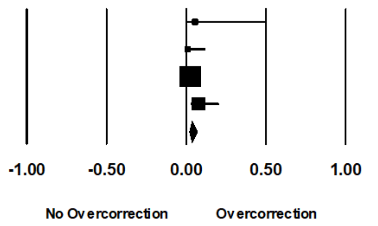

I^2 = 27%

Figure S4 Sensitivity analysis of pooled incidence rates of overcorrection with vaptans

Study name	Event rate	Lower limit	Upper limit	Z-Value	p-Value	Relative weight
Soupart et al (2006)	0.038	0.005	0.228	-3.156	0.002	10.40
Verbalis et al (2011)	0.059	0.019	0.167	-4.659	0.000	24.57
Burst et al (2017)	0.124	0.087	0.174	-9.660	0.000	65.03
	0.092	0.049	0.169	-6.495	0.000	

Statistics for each study; Event rate and 95% CI

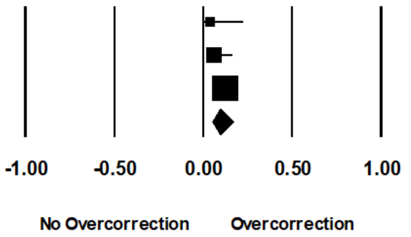

I^2 = 34%

Figure S5 Sensitivity analysis of pooled incidence rates of overcorrection in the control

Study name	Event rate	Lower limit	Upper limit	Z-Value	p-Value	Relative weight
Soupart et al (2006)	0.056	0.003	0.505	-1.947	0.052	2.42
Verbalis et al (2011)	0.008	0.001	0.121	-3.353	0.001	2.54
Burst et al (2017)	0.025	0.016	0.039	-15.695	0.000	95.03
	0.025	0.016	0.039	-16.138	0.000	

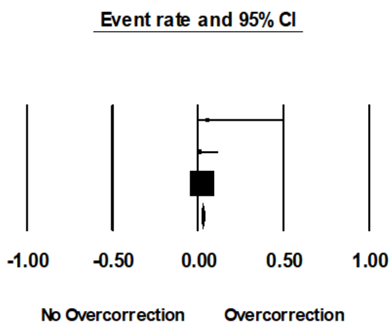

$I^2 = 0\%$

Figure S6 Sensitivity analysis of pooled odds ratio of overcorrection

Study name	Odds ratio	Lower limit	Upper limit	Z-Value	p-Value	Relative weight
Soupart et al (2006)	1.000	0.037	26.934	0.000	1.000	3.12
Verbalis et al (2011)	8.443	0.426	167.487	1.400	0.162	3.80
Burst et al (2017)	5.453	2.983	9.971	5.509	0.000	93.08
	5.258	2.938	9.412	5.588	0.000	

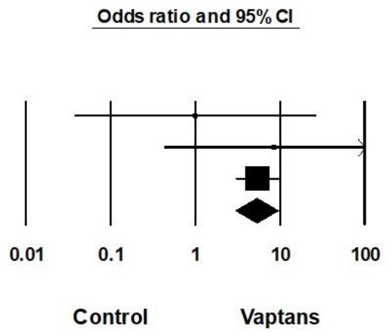

$I^2 = 0\%$

Figure S7 Pooled odds ratio of adverse events requiring vaptan discontinuation

Study name	Odds ratio	Lower limit	Upper limit	Z-Value	p-Value	Relative weight
Soupart et al (2006)	0.667	0.053	8.372	-0.314	0.753	16.83
Verbalis et al (2011)	0.792	0.235	2.669	-0.376	0.707	73.00
Chen et al (2014)	3.585	0.138	92.832	0.769	0.442	10.18
	0.897	0.318	2.533	-0.205	0.838	

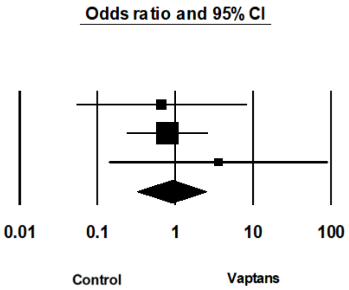

$I^2 = 0\%$

Figure S8 Pooled odds ratio of thirst

Study name	Odds ratio	Lower limit	Upper limit	Z-Value	p-Value	Relative weight
Soupart et al (2006)	1.118	0.042	29.886	0.066	0.947	6.56
Verbalis et al (2011)	2.271	0.708	7.288	1.379	0.168	52.16
Chen et al (2014)	2.850	0.769	10.568	1.566	0.117	41.28
	2.381	1.026	5.526	2.019	0.043	

Odds ratio and 95% CI

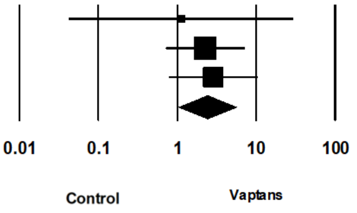

$I^2 = 0\%$

Figure S9 Pooled odds ratio of urinary frequency or polyuria

Study name	Odds ratio	Lower limit	Upper limit	Z-Value	p-Value	Relative weight
Soupart et al (2006)	1.118	0.042	29.886	0.066	0.947	14.85
Verbalis et al (2011)	1.467	0.372	5.788	0.548	0.584	85.15
	1.409	0.397	5.000	0.531	0.595	

Odds ratio and 95% CI

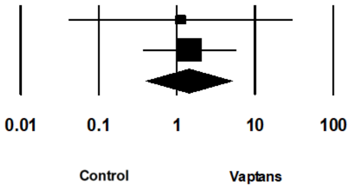

$I^2 = 0\%$

Figure S10 Pooled odds ratio of dry mouth

Study name	Odds ratio	Lower limit	Upper limit	Z-Value	p-Value	Relative weight
Verbalis et al (2011)	1.612	0.519	5.006	0.826	0.409	57.23
Chen et al (2014)	2.850	0.769	10.568	1.566	0.117	42.77
	2.057	0.873	4.847	1.650	0.099	

Odds ratio and 95% CI

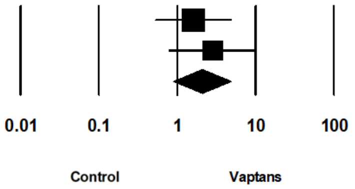

$I^2 = 0\%$

Figure S11 Pooled odds ratio of hypotension

Study name	Odds ratio	Lower limit	Upper limit	Z-Value	p-Value	Relative weight
	Statistics for each study					
Soupart et al (2006)	1.250	0.273	5.733	0.287	0.774	64.58
Verbalis et al (2011)	0.270	0.029	2.498	-1.153	0.249	35.42
	0.726	0.173	3.055	-0.436	0.663	

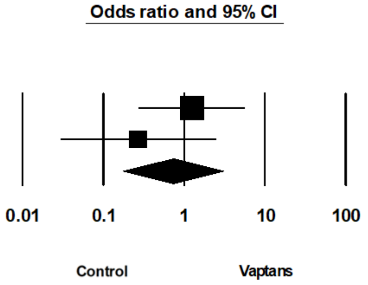

I^2 = 19%

Figure S12 Funnel plot of standard error by difference in mean change of serum sodium from baseline

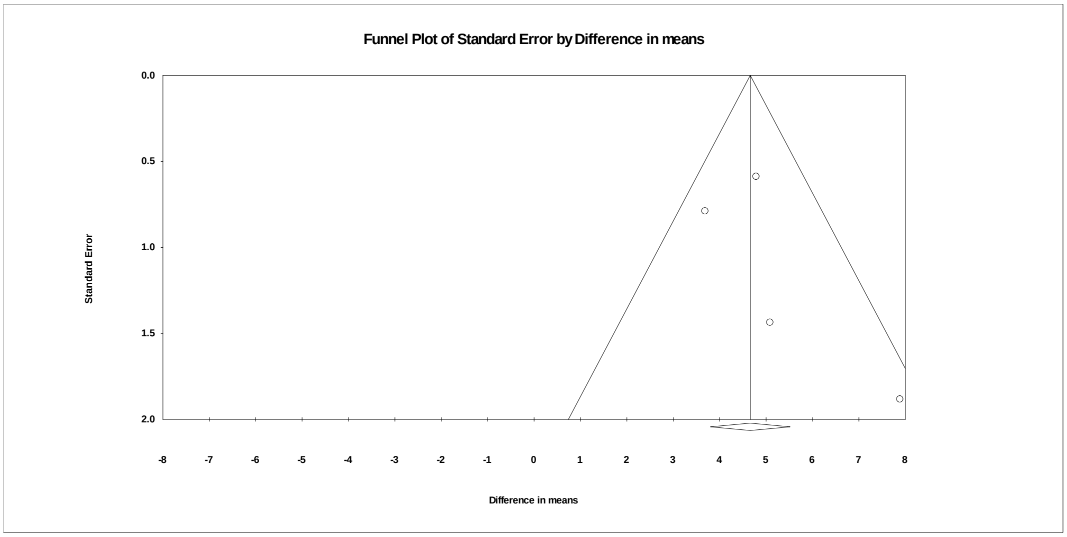

Figure S13 Funnel plot of standard error by log odds ratio of overcorrection

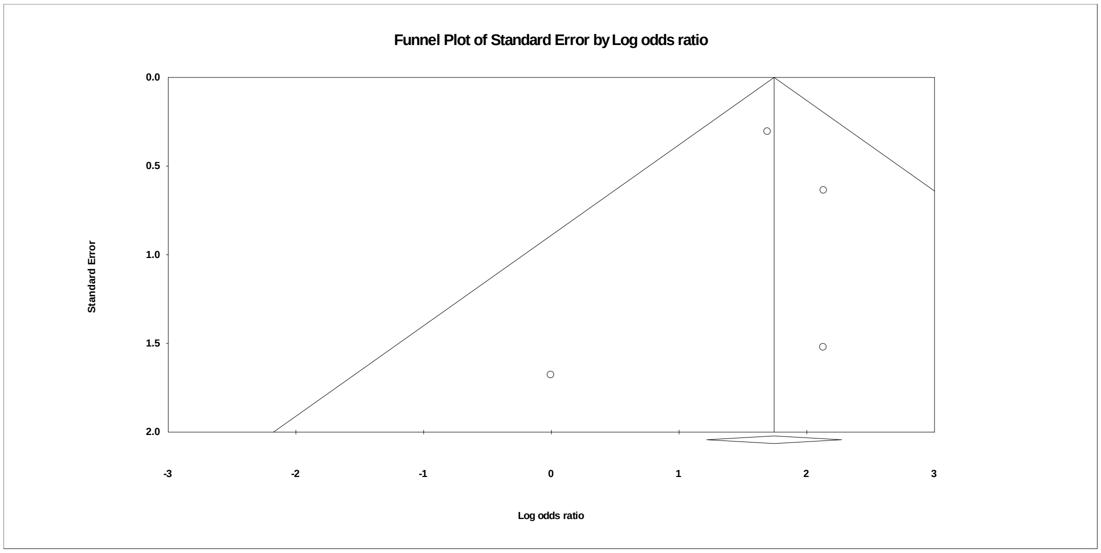
Funnel Plot of Standard Error by Log odds ratio
Standard Error
0.0
0.5
1.0
1.5
2.0
-3
-2
-1
0
1
2
3
Log odds ratio
